# Identification of CD8^+^ T Cell Epitopes in the West Nile Virus Polyprotein by Reverse-Immunology Using NetCTL

**DOI:** 10.1371/journal.pone.0012697

**Published:** 2010-09-14

**Authors:** Mette Voldby Larsen, Alina Lelic, Robin Parsons, Morten Nielsen, Ilka Hoof, Kasper Lamberth, Mark B. Loeb, Søren Buus, Jonathan Bramson, Ole Lund

**Affiliations:** 1 Center for Biological Sequence Analysis, Department of Systems Biology, Technical University of Denmark, Lyngby, Denmark; 2 Department of Pathology and Molecular Medicine, Institute for Molecular Medicine and Health, McMaster University, Hamilton, Ontario, Canada; 3 Division of Experimental Immunology, Institute of Medical Microbiology and Immunology, The Panum Institute, University of Copenhagen, Copenhagen, Denmark; New York University, United States of America

## Abstract

**Background:**

West Nile virus (WNV) is a growing threat to public health and a greater understanding of the immune response raised against WNV is important for the development of prophylactic and therapeutic strategies.

**Methodology/Principal Findings:**

In a reverse-immunology approach, we used bioinformatics methods to predict WNV-specific CD8^+^ T cell epitopes and selected a set of peptides that constitutes maximum coverage of 20 fully-sequenced WNV strains. We then tested these putative epitopes for cellular reactivity in a cohort of WNV-infected patients. We identified 26 new CD8^+^ T cell epitopes, which we propose are restricted by 11 different HLA class I alleles. Aiming for optimal coverage of human populations, we suggest that 11 of these new WNV epitopes would be sufficient to cover from 48% to 93% of ethnic populations in various areas of the World.

**Conclusions/Significance:**

The 26 identified CD8^+^ T cell epitopes contribute to our knowledge of the immune response against WNV infection and greatly extend the list of known WNV CD8^+^ T cell epitopes. A polytope incorporating these and other epitopes could possibly serve as the basis for a WNV vaccine.

## Introduction

*West Nile virus* belongs to the family *Flaviviridae*, along with other human pathogens like *Yellow fever virus* and *Dengue fever virus*. It is an enveloped, spherical virus containing a single strand of RNA that is translated into a continuous polypeptide of approximately 3,400 amino acids. The polypeptide is post-translationally cleaved into ten distinct proteins including three structural proteins; capsid (C) protein, envelope (E) protein, and pre-membrane (prM) protein, and seven non-structural (NS) proteins; NS1, NS2A, NS2B, NS3, NS4A, NS4B, and NS5 [1]. The virus is transmitted to humans by infected mosquitoes and causes West Nile fever in about 20% of infected people. The symptoms of West Nile fever are fever, headache, tiredness, and body aches that can last for a few days to several weeks. Less than one in 100 infected people will develop severe West Nile disease that may lead to fatal encephalitis [2]. The first incidents of WNV infection in the western hemisphere were detected in 1999 during an outbreak of encephalitis in New York City. Since then, the virus has spread across North America and is now a serious threat for public health in the United States, especially for immunocompromised recipients of transplanted organs [1]. Currently, no specific therapy is available for treatment and no vaccine has been approved for prevention of WNV infection in humans [3].

CD8^+^ Cytotoxic T Lymphocytes (CTLs) of the immune system have the capacity to eradicate virus-infected host cells. CTL activation is achieved when peptides originating from virus proteins are presented at the surface of infected cells in complex with Human Leukocyte Antigen (HLA) class I molecules. Several studies have shown that CTLs indeed play a role in the cellular antiviral response against WNV infection in mice and humans [4]–[7].

Although the important role of CTLs in combating WNV is well-established, only a limited number of WNV CD8^+^ T cell epitopes have so far been identified in humans. De Groot et al. applied a bioinformatics approach for predicting HLA-B*07 restricted WNV CD8^+^ T cell epitopes [8]: Out of 16 predicted epitopes, 12 were confirmed to bind HLA-B*07 *in vitro*, but the peptides' ability to induce T-cell responses was not tested. Recent reports from our group and collaborators have described two different strategies for identifying CD8^+^ T cell epitopes in WNV. In the first case, a mass spectroscopy method developed by the Hildebrand laboratory successfully identified four HLA-A*0201 restricted WNV CD8^+^ T cell epitopes [9]. In a second study, we used a shotgun approach, employing overlapping peptides spanning the entire WNV polyprotein and identified additional epitopes restricted by HLA-A*01 and HLA-B*35, as well as several epitopes for which the HLA restriction was not ascertained [10]. In a study by Lanteri et al., overlapping peptides spanning all WNV proteins were likewise tested for their ability to induce T cell responses and led to the discovery of eight frequently recognised WNV peptides [5]. Three of the responses were associated with particular HLA class I types (A*0101, A*0201, and Cw*0303/Cw*0304). In the current study, our objective is to extend the discovery of WNV CD8^+^ T cell epitopes to additional HLA class I alleles, while also considering the sequence variability of WNV proteins. Koo et al. have recently identified regions of the WNV polyprotein that are fully conserved across all analysed WNV sequences and examined whether these regions contain experimentally confirmed or predicted CD8^+^ T cell epitopes [11]. The variability of the WNV proteome is, however, unevenly distributed across the proteome with the structural proteins being most variably. At the amino acid level, the C protein has up to 23% differences across examined sequences, while the NS4b protein has the lowest diversity with at most 8% differences [11]. Accordingly, the majority of the conserved regions identified by Koo et al. were found in the non-structural proteins, while the C protein had none, and the two other structural proteins, prM and E, had the third and fourth least number of conserved regions [11]. It is likely that the structural proteins contain highly immunogenic epitopes that are missed when focusing solely on fully conserved regions. Previous studies have even suggested that the E protein is one of the most immunogenic proteins [5], [10]. It is also possible that the structural proteins experience high variability precisely because it is a selective advantage for the virus to modify these proteins in response to the host immune system. The aim of the present study was therefore to discover novel WNV CD8^+^ T cell epitopes that give a broad coverage of different WNV strains without necessarily being fully conserved across all strains. We employed a two-step bioinformatics reverse-immunology approach: First we used the *NetCTL* method [12], [13] for predicting WNV CD8^+^ T cell epitopes. The *NetCTL* method has previously proven successful in identification of CD8^+^ T cell epitopes in Influenza [14], [15], HIV [16], and Orthopoxvirus [17]. We then selected a subset of the predicted epitopes with a broad coverage of 20 fully-sequenced WNV strains. We were able to confirm that 26 of the predicted epitopes were indeed WNV CD8^+^ T cell epitopes, when tested with a cohort of WNV-infected patients.

## Materials and Methods

### Bioinformatics search strategy for prediction and selection of HLA class I restricted WNV CD8^+^ T cell epitopes

In 2006 when the study was initiated, only 20 WNV polyproteins were available in the GenBank [18] and RefSeq [19] databases (GenBank acc. no. AAM81752.1, AAM81753.1, AAP22088.1, AAP22089.1, AAP22086.1, AAP22087.1, AAQ55854.1, AAR84614.1, AAT02759.1, AAU00153.1, AAV68177.1, AAT95390.1, AAV52687.1, AAV52688.1, AAV52689.1, AAV52690.1, AAW81711.1, AAX09982.1, AAW28871.1, and RefSeq ID NC_001563). Each genome corresponds to a single long polyprotein of approximately 3,400 amino acids. The 20 polyproteins have an average %identity of 96.2% (range 87.0%–99.9%). Using the *NetCTL* method [12], [13] (available at www.cbs.dtu.dk/services/NetCTL), CD8^+^ T cell epitopes were predicted for each of the 12 HLA class I supertypes defined by Lund et al. in [20] (A1, A2, A3, A24, A26, B7, B8, B27, B39, B44, B58, B62). In practice, putative epitopes for a given HLA class I supertype were identified by predicting which peptides are presented by a specific HLA class I allele that represents the entire supertype (for example, HLA-A*0201 represents the A2 supertype, while HLA-A*0101 represents the A1 supertype). In the *NetCTL* method, each nonameric peptide in a protein is assigned a score based on a combination of predictions of proteasomal cleavage, Transporter Associated with antigen Processing (TAP) transport efficiency, and HLA class I affinity. The reliability of *NetCTL* has previously been shown to be as high as or higher than other publicly available methods for CD8^+^ T cell epitope predictions [12], [13]. For predictions of HLA class I affinity, *NetCTL* employs the *NetMHC* method [21], [22], which has been judged to be one of the two best methods in a comparative study of the performance of 30 methods for HLA class I affinity prediction [23]. For each of the 12 HLA class I supertypes and each of the 20 WNV polyproteins, we selected the 17 nonameric peptides with the highest *NetCTL* score (the top 0.5%) as the predicted epitopes. This resulted in a total of 4,080 predicted epitopes of which 484 were unique. To reduce this set, we used the EpiSelect algorithm (previously described in [16]). In short, the EpiSelect algorithm aims, in an iterative procedure, at selecting a given number of predicted epitopes in a way that maximises the coverage of the viral strain with the smallest number of epitopes. Using this algorithm, 16 predicted epitopes were selected for each of the 12 HLA class I supertypes, resulting in a total of 192 peptides. The selected peptides are listed in Supplementary Figure S1 under the reference sequence with RefSeq ID: NC_001563. Note that 17 of the peptides are predicted to be restricted by more than one HLA class I allele, resulting in a total of 175 unique peptides. We are aware that when selecting only a relatively small fraction of the peptides with the highest *NetCTL* scores as the predicted epitopes, we will risk missing some important WNV epitopes. However, due to limited resources, we were not able to test all possible epitopes.

### Bioinformatics methods for identifying possible HLA class I restriction

We investigated to what extent the recognised epitopes could be explained directly in terms of restriction by one of the patient's six HLA class I alleles. For this analysis, the pan-specific *NetMHCpan* prediction method [24], [25] was used. Note that we here use *NetMHCpan*, and not the previously used *NetCTL* method, since the *NetCTL* version that was available when this analysis was performed, only allowed predictions for the 12 HLA class I alleles that represent the 12 HLA supertypes.

It has become apparent that HLA molecules do not present peptides at the same binding threshold [26], [27]. Using a fixed binding affinity threshold would hence result in a bias in the predictions towards HLA molecules with low binding affinity thresholds. Instead, we use the *NetMHCpan* %rank score (previously described in Hoof et al. [28]). The *NetMHCpan* %rank score aims at removing the bias caused by the diverging binding affinity thresholds and placing binding scores for all HLA molecules on an equal scale. In practice, for a given HLA class I molecule, the predicted binding affinity of the identified epitope was ranked along with the predicted binding affinities of a common set of 1,000,000 random, natural, 9meric peptides for the same HLA molecule. A %rank score of, e.g., 5% thus means that only 5% of random peptides are predicted to bind the HLA molecule with an affinity stronger than the identified epitope. The %rank score is calculated for each of the six possible epitope:HLA class I pairs of a patient, and if the lowest %rank score was below 5%, we assigned this HLA class I allele as the restricting HLA, and say that we can successfully explain the epitope restriction. A study by Rao et al. [27] justifies the %rank score threshold of 5%: Rao et al. found that the binding fraction of 9mers among all possible 9mers in the human proteome is ∼5% for HLA-A alleles and ∼2% for HLA-B alleles. Among viral and bacterial proteoms, the binding fraction of peptides is even higher.

### Calculating the epitope conservation

Since initiating the study, additional fully sequenced WNV genomes have become available. For calculating the epitope conservation of the identified epitopes, we examined their frequency in 140 fully sequenced WNV genomes from [11].

### Calculating the epitope coverage

HLA population coverage data was obtained from dbMHC (http://www.ncbi.nlm.nih.gov/gv/mhc/). For each of the 11 epitope:HLA pairs, we first calculated their individual coverage based on the genotype frequency (also called the allele frequency) of the HLA allele and the conservation of the epitope in the 140 examined WNV strains:
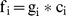
where f_i_ is the coverage of epitope_i_:HLA_i_, g_i_ is the genotype frequency of HLA_i_, and c_i_ is the conservation of epitope_i_.

For each of the three HLA loci (A, B, and C), the coverage can be calculated separately as follows:
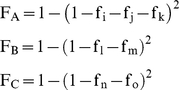
where F_A_, is the coverage of epitope:HLA-A pairs and f_i_, f_j_, and f_k_ are the coverage of the individual epitope:HLA-A pairs.

Where F_B_, is the coverage of epitope:HLA-B pairs and f_l_ and f_m_ are the coverage of the individual epitope:HLA-B pairs.

Where F_C_, is the coverage of epitope:HLA-C pairs and f_n_ and f_o_ are the coverage of the individual epitope:HLA-C pairs.

The total coverage, F, of all 11 epitope:HLA pairs can be calculated as:
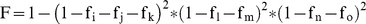
The total coverage was calculated separately for all populations in the following areas: Australia, Europe, North Africa, North America, North-East Asia, Oceania, South America, South-East Asia, South-West Asia, and Sub-Saharan Africa.

### Biochemical peptide-HLA class I binding assay

The biochemical assay for peptide-HLA class I binding was performed as previously described [29], [30]. Briefly, denatured and purified recombinant HLA heavy chains were diluted into a renaturation buffer containing HLA heavy chain, β_2_-microglobulin and graded concentrations of the test peptide, and incubated at 18°C for 48 h allowing equilibrium to be reached. The concentration of generated peptide–HLA complexes was measured in a quantitative enzyme-linked immunosorbent assay and plotted against the concentration of peptide offered [29]. Because the effective concentration of HLA (3–5 nM) used in these assays is below the equilibrium dissociation constant (K_D_) of most high-affinity peptide–HLA interactions, the peptide concentration leading to half-saturation of the HLA is a reasonable approximation of the affinity of the interaction. An initial screening procedure was employed whereby a single high concentration (20,000 nM) of peptide was tested. If no complex formation was found, the peptide was assigned as a non-binder to the HLA molecule in question; conversely, if complex formation was found in the initial screening, a full titration of the peptide was performed to determine the affinity of binding.

### Peptides

Peptides were synthesised as previously described [15]. Briefly, the peptides were synthesised by standard 9-fluorenylmethyloxycarbonyl (FMOC) chemistry, purified by reversed-phase high-performance liquid chromatography (at least 80%, usually >95% purity) and validated by mass spectrometry (Shafer-N, Copenhagen, Denmark). Peptides were distributed at 20 µg/vial and stored lyophilised at −20°C until use. Peptides were dissolved just before use.

### WNV patient study subjects

Thirteen patients infected with WNV were recruited into our study cohort over three seasons (2003–2005) (Table 1). We specifically selected patients who carried HLA-A*0101 or HLA-A*0201 to examine the immunogenicity of the peptides predicted to be restricted by these alleles, since we in our previous report have identified dominant HLA-A*0101 and HLA-A*0201 epitopes [10]. The patients were enrolled following detection of serum WNV IgM (IgM-MAC) by public health laboratories after presenting symptoms of WNV infection. This trial was reviewed and approved by the Research Ethics Board at McMaster University. Written informed consent was obtained from all participants. Serology for WNV and dengue virus was assessed by PRNT as described previously [31]. HLA genotypes were determined using DNA sequence analysis at the Hamilton Health Sciences Histocompatibility Laboratory (Hamilton, ON) and Pure Transplant Solutions (Austin, TX). Blood samples were drawn into heparanised tubes, Peripheral Blood Monocytes (PBMC) were isolated from the blood samples by centrifugation on Ficoll (Amersham Pharmacia) and cryopreserved in RPMI 1640 containing 12.5% human serum albumin (Sigma) and 10% DMSO.

**Table 1:** Characteristics of WNV-infected patients.

Patient ID	Age; sex	Time from onset of symptoms to PBMC collection (days)	HLA-A		HLA-B		HLA-Cw	
44401	54; F	29	0101	0201	0702	1517	0701	0702
44405	65; F	22	0101	0201	0702	1501	0303	0702
55302	65; M	32	01	02	57	40	ND	ND
55307	55; F	40	0101	0301	3701	4429	0501	0602
55308	33; F	55	0101	0301	0801	4701	0602	0701
55309	64; F	120	0201	0301	3503	4403	0401	0401
55310	64; F	31	0101	0101	0801	0801	0701	0701
55405	47; F	73	0101	0301	0702	0801	0701	0702
55407	63; M	66	0101	0301	1302	3503	0401	0602
55410	51; M	93	0201	0201	4001	4402	0304	0501
55413	51; M	120	0101	0201	0801	4402	0701	0501
55414	57; M	135	0201	3101	0702	5601	0102	0702
55415	43; F	136	0201	0201	2702	5601	0102	0202

Note that the HLA-A and -B alleles of patient #55302 were only determined by low-resolution serological typing. ND: Not Determined.

### IFN-γ enzyme-linked immunosorbent spot (ELISPOT) assay

PBMCs were screened in an initial IFN-γ ELISPOT assay to demonstrate peptide reactivity without *a priori* knowledge of patient HLA types. 112 putative epitopes with measured K_D_<500 nM were assembled into 20 peptide pools according to a 2-D grid, where each peptide was present in only two pools. Coincident reactivity between two pools identified candidate peptides containing putative T cell epitopes. T cell reactivity was subsequently validated by restimulation of PBMCs from the same patient with individual peptides. IFN-γ ELISPOTs were performed using kits purchased from BD Biosciences and carried out according to the manufacturer's instructions. PBMCs were thawed and placed immediately into cRPMI pre-warmed to 37°C. The cells were aliquoted into the ELISPOT plate at 1–2×10^5^ cells/well and peptides were added at a final concentration of 2 µg/ml per peptide. The plates were incubated for 18 to 20 hours at 37°C in a humidified incubator with 5% CO_2,_ and the assay was completed according to the manufacturer's directions. Spots were enumerated using an ImmunoSpot 3B analyser (Cellular Technology Ltd, Cleveland, OH). Positive reactivity was defined as responses that were at least two-fold above background and a minimum of 50 SFC/10^6^ PBMCs. As a positive control for CD8^+^ T cell reactivity, all samples were stimulated with a collection of WNV-specific CD8^+^ T cell epitopes that was previously found to be frequently recognised in any given patient [10]. This pool of peptides is termed “pool of dominant WNV epitopes” in the text and consists of the following sequences: SGATWVDLV, SVGGVFTSV, WMDSTKATRY, SLFGQRIEV, MPNGLIAQFY, GTKTFLVHREWFMDL, FLVHREWFMDLNLPW, LGLQKLGYILREV, DTAGWDTRITRADL. Note that we here use the term *dominant* to describe epitopes that are frequently recognised in any given patient (as opposed to epitopes that elicit a strong immune response). None of the peptides in the WNV peptide pool were also in the set of predicted, selected epitopes described in the subsection *Bioinformatics search strategy for prediction and selection of HLA class I restricted WNV CD8^+^ T cell epitopes*.

### ICS validations

Intracellular cytokine staining (ICS) was employed to confirm that IFN-γ secreting cells identified by ELISPOT were actually CD8^+^ T cells. Given the limiting amount of patient material available to our group, we chose to employ a recently described method for unbiased amplification of CD8^+^ T cells to expand our frozen PBMCs prior to analysis [32]. Briefly, K64-4-1BBL cells were loaded with anti-CD3 and anti-CD28 and irradiated at 10,000 rads. Freshly thawed PBMCs were incubated with the loaded, irradiated K64-4-1BBL cells at a ratio of 2∶1. We routinely observed 5 to 10 fold expansion in CD8^+^ T cell numbers in the period of 10 to 12 days. The cultures were subsequently collected, washed, and used immediately for ICS. This initial, unbiased expansion step greatly increases the number of CD8^+^ T cell effectors capable of recognising specific epitopes. Most importantly, this method does not alter the hierarchy of epitope reactivity (Supplementary Figure S2). Therefore, this method allowed us to both confirm the specificity of the epitope and define the reactivity as dominant or subdominant in terms of magnitude of response.

The ICS protocol was conducted as previously described [10] with some modifications. Briefly, cells were aliquoted (1–2×10^6^ cells/well) into 96-well U-bottomed plates. Peptides were added to a final concentration of 2 µg/ml and the cells were incubated for 2 hours. Brefeldin A was then added to a final concentration of 5 µM and the cells were incubated 4 hours further. At the end of this period, cells were pelleted and washed in 10 µM EDTA. The cells were subsequently surface stained with either anti-CD8-PE-Cy7 or anti-CD3-PE-Cy5, permeabilised with Cytofix/Cytoperm and intracellular cytokines were identified using anti-TNF-α-PE and anti-IFN-γ-APC (Note: All flow cytometry reagents were obtained from BD Pharmingen). Fluorescence data were acquired using a FACSCanto or an LSRII and 200,000 events based on the live lymphocyte gate were collected per sample.

## Results

### Prediction and selection of HLA class I restricted CD8^+^ T cell epitopes

To identify WNV CD8^+^ T cell epitopes with a broad coverage of WNV strains, we first used the *NetCTL* method [12], [13] to predict HLA class I supertype restricted epitopes. We then selected a subset of 175 predicted epitopes that constitutes broad coverage of 20 WNV strains as described in *Materials and Methods*. Of the 175 predicted epitopes, 14 could not be synthesised. To determine whether the remaining 161 peptides were indeed binders to the relevant HLA class I molecules, they were tested in a biochemical *in vitro* binding assay. Overall, 112 peptides (70%) had a binding affinity (K_D_) of 500 nM or less for the relevant HLA class I molecule (Supplementary Table S1). It has previously been shown that the vast majority of HLA class I restricted epitopes bind their relevant HLA molecule with a K_D_ of 500 nM or less [33].

### Immunogenicity of the predicted epitopes

**Figure 1:**
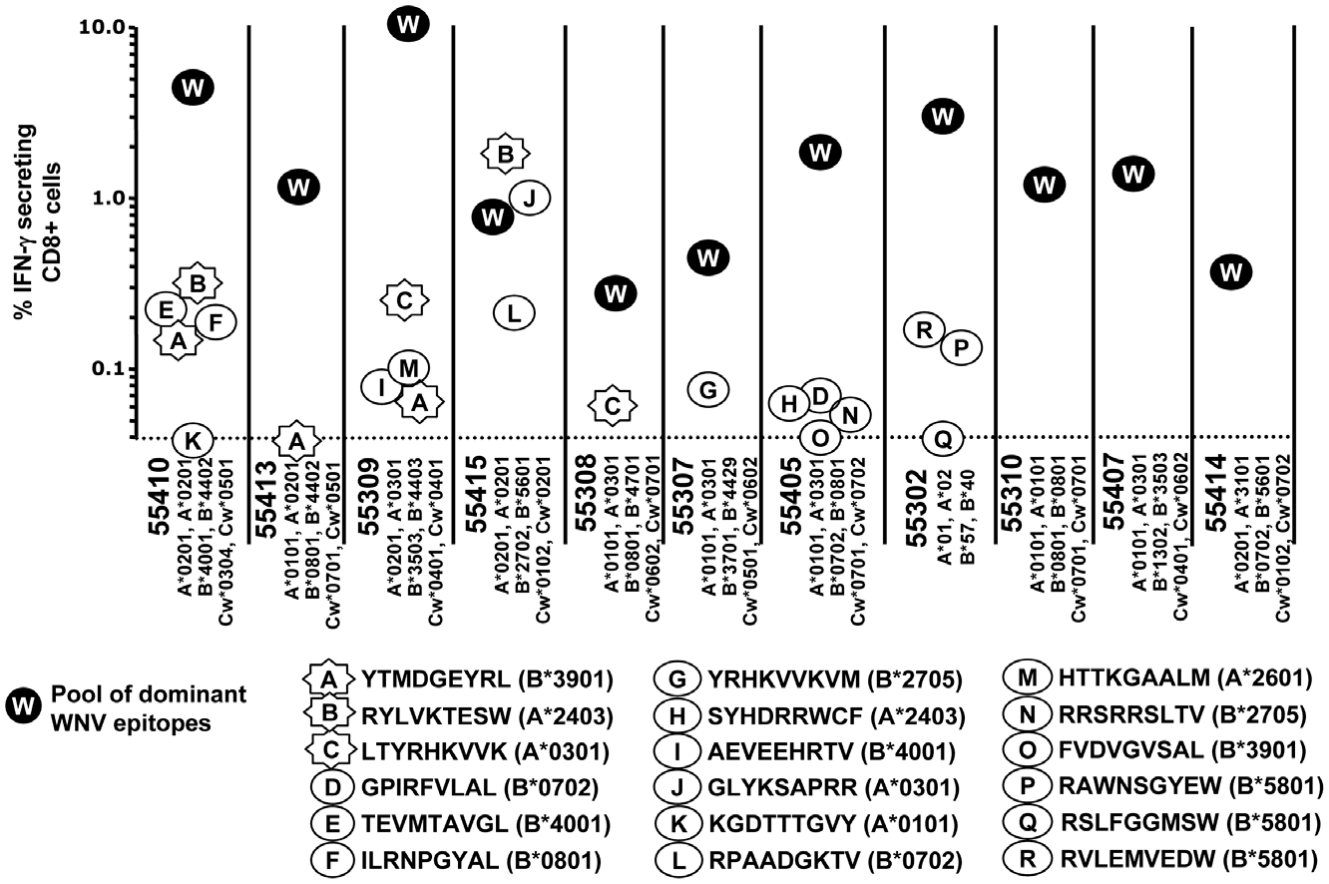
Immunogenicity of the predicted epitopes. One-hundred-and-twelve predicted epitopes were screened for reactivity by IFN-gamma ELISPOT using samples from 11 patients. Patient ID is listed on the X-axis along with the HLA alleles of each patient. Each circle/star represents an individual peptide. Circles correspond to peptides that displayed reactivity in only one patient. Stars correspond to peptides that displayed reactivity in more than one patient. As a positive control for these analyses, a pool of nine dominant WNV epitope peptides (labeled W) was included in each analysis.

In the first round of analysis, the 112 peptides identified as binding with a K_D_ of 500 nM or less for the relevant HLA class I molecule, were tested for their ability to stimulate CD8^+^ T cells from a study population of 11 WNV-infected patients. As shown in Figure 1, we were able to confirm that 18 predicted epitopes were recognised by CD8^+^ T cells from these naturally-infected patients.

As a positive control, we used a pool of WNV epitopes that we have previously found to be recognised frequently in any given patient [10]. We denote this peptide collection the “pool of dominant WNV epitopes”. Figure 1 shows that the positive control generally evoked a higher T cell response than the individual epitopes, which is not surprising, since the positive control contained *nine* previously identified WNV epitopes. Eight of the eleven patients exhibited reactivity to at least one of the predicted epitopes. These responses were typically subdominant in terms of magnitude of response, with the exception of RYLVKTESW and GLYKSAPRR in patient #55415. Three epitopes (Figure 1; star shaped) were recognised by more than one patient.

**Figure 2:**
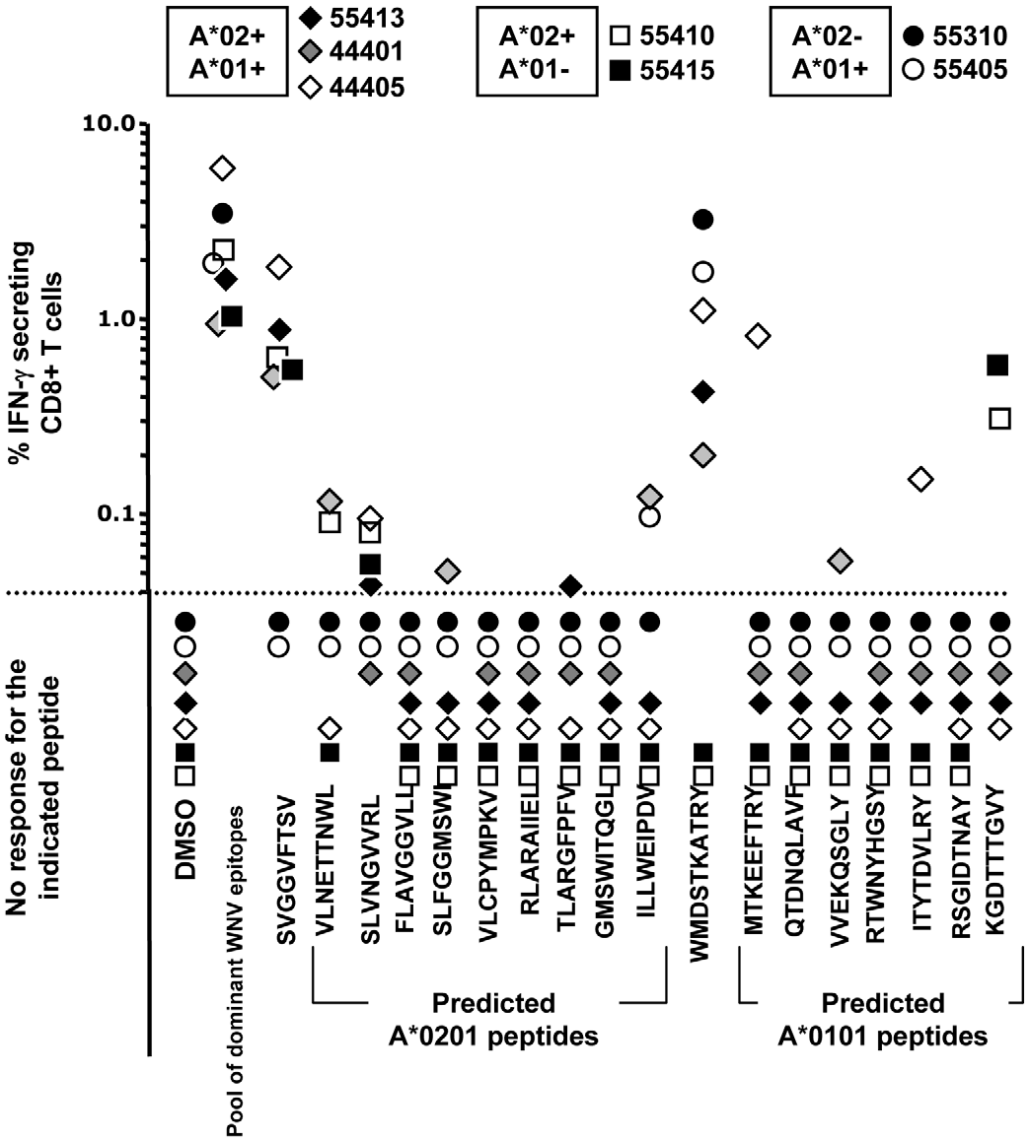
Immunogenicity of predicted HLA-A*0201 and HLA-A*0101 epitopes. Nine predicted epitopes confirmed to bind HLA-A*0201 and seven predicted epitopes confirmed to bind HLA-A*0101 were screened for reactivity by ICS assay using samples from seven patients that expressed either HLA-A*0201, HLA-A*0101, or both (listed on the X-axis). Each dot represents an individual patient. Patients expressing both HLA-A*0201 and HLA-A*0101 are represented by diamonds; patients expressing only HLA-A*0201 are represented by squares; patients expressing only HLA-A*0101 are represented by circles. Test peptides are listed on the X-axis. As a positive control for these analyses, a pool of nine dominant WNV epitopes (labeled W) was included in each analysis. Furthermore, SVGGFTSV is a dominant epitope restricted by HLA-A*0201, while WMDSTKATRY is a dominant epitope restricted to HLA-A*0101. Please note that the part of the graph below the dotted line consists of donors with no response to the indicated peptides.

It is possible that technical complications resulting from the peptide pooling method may have obscured reactivity towards some putative epitopes. To address this possibility, we carried out a second round of analysis. This time we used only the peptides predicted to bind HLA-A*0101 or HLA-A*0201 because our previous study [10] had revealed that both of these alleles present epitopes (WMD10 = WMDSTKATRY restricted by HLA-A*0101 and SVG9 = SVGGVFTSV restricted by HLA-A*0201) that are both dominant with regards to frequency of recognition (they were recognised in all HLA-matched patients) and magnitude of response. Accordingly, the seven patients carrying either HLA*0101, HLA*0201, or both provided us with a robust method of characterising the predicted epitopes relative to previously defined epitopes. Two of the seven patients (patient #44401 and #44405) had not been tested in the first round of analysis. As seen in Figure 2, all patients possessed CD8^+^ T cell reactivity to the pool of dominant WNV epitopes. Furthermore, all patients carrying the HLA-A*0101 allele exhibited robust reactivity to WMD10 (WMDSTKATRY) and all patients carrying HLA-A*0201 exhibited reactivity to SVG9 (SVGGVFTSV).

The analysis did unveil some reactivities that were not identified in the first round of analysis. Five of the nine HLA-A*0201 peptides and four of the seven HLA-A*0101 peptides evoked some degree of reactivity in the patient cohort. For the most part, responses to these peptides were subdominant both in terms of magnitude of response and in frequency of recognition (none of the peptides were recognised uniformly by all of the patients in our cohort). Two HLA-A*0101-binding peptides, MTKEEFTRY and KGDTTTGVY, were recognised at levels comparable to the known epitope WMD10 (WMDSTKATRY). Surprisingly though, peptide KGDTTTGVY only stimulated responses in patients who were HLA-A*0101-negative.

Compiling the results presented in Figure 1 and 2, three patients (#55310, #55407, and #55414) had no response to any of the tested peptides. Two patients (#55307 and #55308) each only responded against one of the tested peptides, while the highest number of responses was found using PBMC from patient #55410: Here, responses against seven different epitopes were detected. The average number of responses per patient was 3.6. For clarity, Supplementary Table S2 compiles the results from Figure 1 and 2 and lists them per identified epitope. In total, 26 epitopes were identified. They gave rise to 36 responses in ten WNV infected patients.

None of the identified epitopes induced a response in all patients expressing the predicted restricting HLA class I allele. At most, any individual epitope elicited a response in four different patients. For instance, VLNETTNWL-A*0201 was stimulatory for CD8^+^ T cells from patients #44401 and #55410, but not from #55413, #44405, or #55415, even though all five patients carry HLA-A*0201. The lack of conservation of epitopes in different WNV strains may explain some of these observations. For instance, the epitope AEVEEHRTV is only found in 8% of the 140 fully sequenced WNV strains, which might explain why patient #55410 did not exhibit a response to this peptide, although he carry the predicted restricting HLA class I allele, HLA-B*4001. However, this argument cannot explain all of our observations. As an example, the HLA-A*0301 restricted epitope LTYRHKVVK is found in all 140 examined WNV strains, but did not evoke a response in patient #55307, #55405, or #55407, although these patients all express HLA-A*0301.

### Distribution of the epitopes

**Figure 3:**
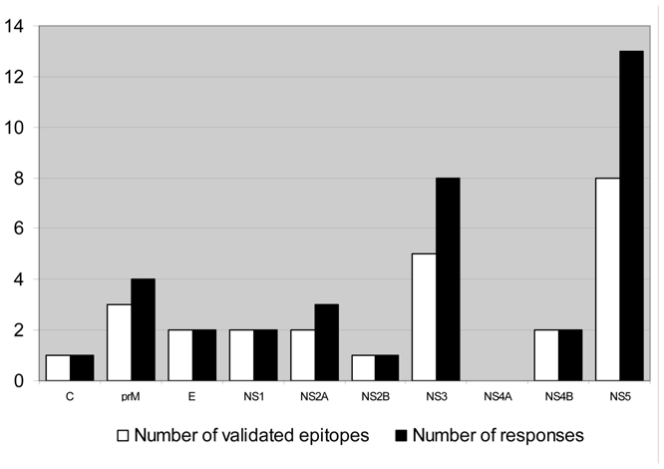
Location of the identified epitopes. The figure shows the number of validated epitopes in each of the ten WNV proteins and the number of responses that they evoked. C: Core protein, prM: Membrane protein, E: Envelope protein, NS1: Non-structural protein 1, NS2A: Non-structural protein 2A, NS2B: Non-structural protein 2B, NS3: Non-structural protein 3, NS4A: Non-structural protein 4A, NS4B: Non-structural protein 4B, NS5: Non-structural protein 5.

The identified epitopes and T cell responses are distributed across the WNV proteins as illustrated in Figure 3.

Most validated epitopes were found in the NS3 and NS5 proteins, which are indeed the two longest proteins containing the highest number of tested predicted epitopes (34 for NS3 and 50 for NS5). Epitopes in these proteins likewise gave rise to most T cell responses. No epitopes were found in NS4A, which is one of the shortest proteins with only five tested predicted epitopes. Six of the epitopes were found in structural proteins including one in the C protein, three in prM, and two in the E protein. Based on the present study, we were not able to observe a particular bias towards epitope location in certain WNV proteins, besides what can be explained by the mere size of the protein. We were thus not able to confirm findings of other studies [5], [10] that protein E is more commonly targeted by T cell responses.

### Suggested HLA class I restriction

Table 2 lists the 36 observed epitope responses identified in Figure 1 and 2 and compares them with the HLA class I types of the patients as listed in Table 1.

**Table 2:** Predicted HLA class I restriction of the 36 observed responses.

Patient	Sequence	Selecting HLA	Restricting HLA	%Rank	Direct HLA match	Super-type match	Explainable by %rank
44401	ILLWEIPDV	A*0201	A*0201	0.30	X	X	X
44401	VLNETTNWL	A*0201	A*0201	1.00	X	X	X
44401	SLFGGMSWI	A*0201	A*0201	0.80	X	X	X
44401	VVEKQSGLY	A*0101	A*0101	0.15	X	X	X
44405	**MTKEEFTRY**	A*0101	A*0101	0.40	X	X	X
44405	SLVNGVVRL	A*0201	A*0201	3.00	X	X	X
44405	ITYTDVLRY	A*0101	A*0101	0.10	X	X	X
55302	RAWNSGYEW	B*5801	B*57	NA		X	NA
55302	RSLFGGMSW	B*5801	B*57	NA		X	NA
55302	RVLEMVEDW	B*5801	B*57	NA		X	NA
55307	**YRHKVVKVM**	B*2705	Cw*0602	1.50			X
55308	**LTYRHKVVK**	A*0301	A*0301	0.30	X	X	X
55309	**AEVEEHRTV**	B*4001	B*4403	1.50		X	X
55309	HTTKGAALM	A*2601	NA	6.00			*
55309	LTYRHKVVK	A*0301	A*0301	0.30	X	X	X
55309	YTMDGEYRL	B*3901	A*0201	1,00			X
55405	FVDVGVSAL	B*3901	B*0702	3,00			X
55405	**GPIRFVLAL**	B*0702	B*0702	0.30	X	X	X
55405	ILLWEIPDV	A*0201	NA	32.00			
55405	**RRSRRSLTV**	B*2705	B*0801	4.00			X
55405	SYHDRRWCF	A*2403	B*0801	3.00			X
55410	SLVNGVVRL	A*0201	A*0201	3.00	X	X	X
55410	**ILRNPGYAL**	B*0801	Cw*0304	1.50			X
55410	KGDTTTGVY	A*0101	NA	32.00			**
55410	RYLVKTESW	A*2403	NA	6.00			
55410	**TEVMTAVGL**	B*4001	B*4001	0.15	X	X	X
55410	VLNETTNWL	A*0201	A*0201	1.00	X	X	X
55410	**YTMDGEYRL**	B*3901	A*0201	1.00			X
55413	YTMDGEYRL	B*3901	A*0201	1.00			X
55413	SLVNGVVRL	A*0201	A*0201	3.00	X	X	X
55413	TLARGFPFV	A*0201	A*0201	0.10	X	X	X
55415	KGDTTTGVY	A*0101	NA	32.00			
55415	SLVNGVVRL	A*0201	A*0201	3.00	X	X	X
55415	GLYKSAPRR	A*0301	NA	50.00			
55415	**RPAADGKTV**	B*0702	B*5601	0.80		X	X
55415	**RYLVKTESW**	A*2403	B*2702	0.80			X

The columns lists: **Patient:** Patient ID, **Sequence:** Epitope amino acid sequence, **Selecting HLA:** The HLA class I allele used for selecting the epitope, **Restricting HLA:** The HLA class I allele by which the epitope is predicted to be restricted in this patient using the *NetMHCpan* method. NA indicate that none of the patient's HLA molecules were predicted to present the peptide with a %rank score less than or equal to 5. **%rank:** The rank of the epitope among 1,000,000 random, natural, 9meric peptides based on the predicted binding affinities to the restricting HLA, **Direct match:** The patient carries the HLA class I allele for which the epitope is selected, i.e., the selecting and restricting HLA class I alleles are identical. **Supertype match:** The patient does not carry the HLA class I allele for which the epitope is selected, however, the selecting and restricting HLA class I allele belong to the same HLA class I supertype. **Explainable by %rank:** The patient does not carry the HLA class I allele for which the epitope is selected, but another HLA class I allele, which is also predicted to present the epitope (the %rank value for the restricting HLA is below 5.00).

Note that the rank-analysis was not performed for patient #55302, since the HLA-A and -B alleles of this patient was only determined by low-resolution serological typing and the HLA-C alleles are undetermined. The three epitopes that are recognised in this patient are, however, all well presented by B*5701 (by serological typing it is known that patient #55302 carries B57) with %rank scores between 0.1% and 0.2%.

*This peptide can be presented as an 8mer, HTTKGALL, to B*3503 with a %rank score of 5%.

**This peptide can be presented as an 8mer, GDTTTGVY, by B*4402 with a %rank score of 5%.

Epitopes marked in bold are used for the population coverage calculations.

Almost half (16 out of 36) of the observed responses can be explained by a direct match between the patient HLA class I type and the HLA class I allele used for selecting the given epitope (see Table 2). This result reflects that we started out by predicting epitopes restricted by the 12 HLA class I alleles that represent the major class I supertypes [20], but tested the predicted epitopes in all patients, regardless of whether the patients carried any of these specific 12 HLA class I supertype representative alleles or, for example, another HLA class I allele belonging to the same supertype. In accordance with this, the fraction of explainable epitope responses improved to 58% (21 out of 36), when also considering HLA class I supertype matches between HLA class I alleles expressed by the patients and the HLA class I alleles used for selecting the epitopes. For instance; AEVEEHRTV was selected for binding to HLA-B*4001, which represents the B44 supertype, but induced a response in patient #55309, who does not carry HLA-B*4001. However, patient #55309 carry HLA-B*4403, which is also a member of the B44 supertype. Likewise, RPAADGKTV was selected for binding to HLA-B*0702, but induced a response in patient #55415, who does not carry HLA-B*0702, but HLA-B*5601, an allele belonging to the B7 supertype [20].

For some of the identified epitopes there was more often a complete match between the HLA class I allele used for selecting the epitope and the HLA class I type of the patient displaying the response. For instance, nine of the epitopes that were predicted to be presented by the representative of the A2 supertype, HLA-A*0201, induced a response in HLA-A*0201-positive patients. In contrast, none of the epitopes that were predicted to be presented by the representative of the B39 supertype, HLA-B*3901, induced a response in B*3901-positive patients for the simple reason that none of the patients carry HLA-B*3901.

Since not all responses can be explained in terms of a direct match between the HLA class I allele used for selecting the epitope and the HLA class I alleles carried by the patient, nor by the supertype association of one of the HLA class I alleles carried by the patient, an alternative approach for identifying the most likely restricting allele in each responding patient was applied. We used a pan-specific peptide:HLA binding prediction algorithm called *NetMHCpan* [24], [25] for investigating whether the recognised epitopes could be explained in terms of binding to one of the patient HLA class I alleles. Note that the *NetCTL* method [12], [13], which was used for the initial epitope predictions, could not be used for this analysis, since *NetCTL* only allows predictions for the 12 HLA class I alleles that represent the 12 HLA class I supertypes. The summary of the analysis is shown in Table 2. The analysis did not include the three responses detected in patient #55302, since the HLA types of this patient were only determined by low-resolution serotyping. In short, the analysis was performed by calculating the *NetMHCpan* %rank score for each of the six possible epitope:HLA class I pairs as described in *Material and Methods*. If the lowest %rank score was below 5%, we assigned the HLA class I allele that resulted in this score as the restricting HLA and say that we can successfully explain the epitope restriction. Using this definition, we assigned 82% (27 out of 33, see Table 2) of the detected epitope specific T cell responses to a specific HLA class I allele.

As seen in Table 2, six responses remain unexplainable. For instance, ILLWEIPDV was selected for binding to HLA-A*0201, but induced a response in patient #55405, who does not express HLA-A*0201. Among the HLA class I alleles of #55405, HLA-A*0301 resulted in the lowest %rank score, 32, but this is well over the defined threshold of 5%. However, two of these six responses can be explained in terms of nested 8mer peptides. For instance, the 8mer peptide GDTTTGVY nested within KGDTTTGVY is predicted to bind within the 5% rank to the HLA-B*4402 allele.

Disregarding the six cryptic restrictions mentioned above including the nested peptide restrictions, we suggest that the 26 identified WNV CTL epitopes are restricted by 11 different HLA class I alleles (A*0101, A*0201, A*0301, B*0702, B*0801, B*2702, B*4001, B*4403, B*5601, Cw*0304, Cw*0602) covering 7 of the 12 major HLA-A and HLA-B supertypes. Table 3 lists the genotype frequency of these alleles in different areas of the world.

**Table 3:** Allele frequencies of 11 HLA class I alleles in different areas of the world.

HLA	Australia	Europe	North-East Asia	North America	Oceania	South-East Asia	South-West Asia	South America	Sub-Saharan Africa	North Africa
A*0101	0,022	0,164	0,059	0,042	0,003	0,007	0,094	0,002	0,056	0,137
A*0201	0,127	0,272	0,153	0,145	0,144	0,069	0,158	0,221	0,103	0,176
A*0301	0,014	0,141	0,037	0,037	0,005	0,006	0,048	NA	0,051	0,040
B*0702	0,011	0,139	0,054	0,038	0,003	0,007	0,026	0,006	0,044	0,029
B*0801	0,012	0,118	0,004	0,022	NA	0,003	0,043	NA	0,042	0,077
B*2702	NA	0,005	0,001	0,002	NA	NA	0,001	NA	NA	0,007
B*4001	0,092	0,049	0,045	0,022	0,149	0,165	0,007	0,002	0,004	0,007
B*4403	0,001	0,049	0,047	0,018	0,005	0,015	0,029	NA	0,035	0,099
B*5601	0,161	0,004	0,005	0,004	0,005	0,016	0,001	NA	0,003	NA
Cw*0304	0,009	0,065	0,098	0,218	0,108	0,173	0,009	0,238	0,048	NA
Cw*0602	0,009	0,091	0,086	0,042	0,005	0,014	0,117	0,002	0,145	NA

HLA population coverage data was obtained from dbMHC (http://www.ncbi.nlm.nih.gov/gv/mhc/).

NA: Not available.

We are aware that the suggested restricting HLA class I alleles represent only the most likely restricting element, and that these assignment are merely based upon predictions.

### Population coverage

Since a key objective of this study is to identify CD8^+^ T cell epitopes that collectively have a broad coverage of WNV strains and thereby are of particular interest for vaccine development, we next examined the theoretical population coverage in different areas of the World with a minimal epitope set consisting of the 11 epitopes marked in bold in Table 2. These 11 epitopes were selected because they each are restricted by one or two of the 11 suggested restricting HLA class I alleles. If more than one epitope could be selected for the same HLA class I allele, we chose the more conserved epitope. Although additional WNV epitopes are known from previous studies [5], [9], [10] and others are likely still undiscovered, the analysis illustrates the coverage that could be obtained by a small set of epitopes.

We hypothesise that although we could not detect a response against all epitopes in all HLA class I matched patients even for 100% conserved epitopes, *immunising* with the epitopes will lead to CD8^+^ T cell activation in all HLA class I matched individuals. This hypothesis is supported by a study by Assarsson et al., where CD8^+^ T cell responses were detected against all previously identified epitopes after immunising transgenic mice with the epitopes [34]. Whether or not the memory CD8^+^ T cells will later recognise cells infected with WNV depends on which epitopes the infecting WNV strain contain. The population coverage of the 11 epitope:HLA class I pairs is accordingly calculated by considering both the HLA class I allele frequencies and the epitope conservation as described in *Material and Methods*. Table 4 summarises the coverage of the 11 epitope:HLA class I allele pairs in ten areas of the World. Considering only the 11 restricting HLA class I alleles identified in this study, more than half of the population is covered in nine out of the ten areas of the World. For the North American population the coverage is 72%, while the coverage is 93% for the European population. The smallest coverage is found in Australia, where 48% of the population is covered.

**Table 4:** Epitope coverage in ten areas of the World.

Area	Coverage
Australia	0.48
Europe	0.93
North Africa	0.67
North America	0.72
North-East Asia	0.69
Oceania	0.59
South America	0.65
South-East Asia	0.62
South-West Asia	0.67
Sub-Saharan Africa	0.66

HLA class I allele frequencies were obtained from dbMHC (http://www.ncbi.nlm.nih.gov/gv/mhc/). Coverage is calculated as described in the subsection *Calculating the epitope coverage* in *Materials and Methods*. A Coverage of 1 corresponds to maximum (full) coverage.

## Discussion

Using reverse immunology and employing bioinformatics methods, we have discovered 26 new WNV specific CD8^+^ T cell epitopes, which significantly extends the repertoire of known WNV CD8^+^ T cell epitopes. We suggest that the newly discovered epitopes are restricted by 11 different HLA class I alleles.

When we initiated our study, only 20 fully-sequenced genomes from WNV strains were publically available, and they form the basis of our predictions. Since then, additional WNV strains have been sequenced and the WNV variability has been analysed at a larger scale [11]. Our approach included selecting predicted WNV epitopes that experience broad coverage of the 20 originally sequenced WNV strains. It is likely that we would select a different set of broadly covering predicted epitopes, if we were to repeat the study using data from all presently available WNV strains. Nevertheless, our results indicate that selecting predicted epitopes with a broad coverage of WNV strains - in contrast to 100% conserved epitopes - enables identification of more epitopes in the structural WNV proteins. These proteins vary the most and hence contain the fewest fully conserved regions [11].

In the present study, we observed that the number of predicted epitopes is a direct function of protein size. However, in our recent study, we observed that the interindividual patterns of CD8^+^ T cell dominance (the frequency of recognition) do not correlate with protein size but rather with the individual's HLA. As an example, individuals expressing HLA-A*0201 were primarily reactive to an epitope in E and an epitope in NS4b [9], [10], while individuals expressing HLA-A*0101 displayed a CD8^+^ T cell response directed against prM. Thus, protein size alone is not sufficient to explain dominance within individuals or between individuals. Furthermore, we and our collaborators have recently reported a direct survey of WNV peptides bound by HLA-A*0201 in infected cells [9] and did not observe a correlation between protein size and natural loading of HLA class I. It should be noted that we identified a number of epitopes in the present study that evoked more robust responses in some of the patients than were observed with our previously identified collection of “dominant” epitopes. These observations highlight the complexities of antigen processing and stress the importance of using combined methodologies (*in silico*, *in vitro*, and *in vivo*) for epitope discovery.

The complex epitope recognition pattern in the WNV infected patients showed that not all peptides that induce a CTL response in one patient do so in all patients expressing the restricting HLA class I allele. This is true even for epitopes that are fully conserved across all analysed WNV strains. It is, however, inevitable that not all patients expressing the appropriate HLA allele will respond to a given epitope restricted by this allele due to factors like dominance, competition, “holes” in the T cell repertoire etc. In fact, in a recent work we show that only 34–50% of patients expressing an appropriate HLA allele will respond to an epitope restricted by this allele [35]. The fact that not all patients expressing a given allele respond to all epitopes restricted by this allele is thus not an indication of a faulty prediction method, but rather a result of factors we cannot control.

Unlike the results of our recent study identifying WNV CD8^+^ T cell epitopes, where reactivity to four dominating epitopes were found in almost all patients expressing the restricting HLA class I allele [10], the CD8^+^ T cell epitopes identified in the present study maximally induced response in about 25% of patients bearing the appropriate HLA. It seems that the CD8^+^ T cell response against WNV includes both a few epitopes recognised in the majority of infected individuals - interindividually dominant epitopes - as well as a broad response against interindividually subdominate epitopes that each are recognised in some infected individuals, but not in others. Similar observations are apparent for other small RNA viruses, e.g., Influenza A virus: Almost all HLA-A*0201 positive individuals were found to respond against the epitope M1_58–66_ in a study from 1995 [36], while CD8^+^ T cell epitopes identified in a later study were responsive in only some patients carrying the restricting HLA class I alleles [15], [37]. CD8^+^ T cell responses against HIV have also been found to contain both interindividually dominant and subdominant epitopes [16], [38], [39].

We tested all peptides with an *in vitro* determined HLA class I binding affinity below (i.e. better than) 500 nM in all the WNV infected patients. Half of the responses were found in patients not expressing the predicted restricting HLA class I allele. The concordance between predictive and actual HLA class I restriction could be slightly improved by taking into account the supertype association of the patient HLA class I alleles. In contrast, only 18% (six responses) remained unexplainable when applying a pan-specific HLA peptide binding prediction method for calculating the %rank score of the epitope to each of the responding patient's six HLA class I alleles and considering the allele with a %rank score below 5 as the restricting allele. These results confirm recent findings that HLA class I supertypes often provide an oversimplification of the HLA class I specificity space [24], [40], [41]. Moreover, and maybe more importantly, this analyses shows that the majority of these immune responses are indeed predictable using advanced bioinformatics methods for pan-specific HLA-peptide binding and that cellular responses are hence directly explained in terms of peptide binding to one of the patients HLA molecules in accordance with earlier work by for instance Hoof et al. [28].

Despite the complex epitope recognition pattern observed, we hypothesised that all of the newly identified WNV epitopes will induce a CTL response in all individuals carrying the restricting HLA class I allele, if the individuals were to be *immunised* with the epitopes. This hypothesis is supported by a study concerning the repertoire of CD8^+^ T cell epitopes recognised after Vaccinia Virus infection [34]. Here it is shown that all Vaccinia Virus CD8^+^ T cell epitopes identified in a previous study in the context of natural infection [42] were able to elicit CTL responses in mice immunised with the epitopes. Similar immunological analysis is required to verify that the WNV epitopes identified in the present study are able to induce a successful antiviral response in a host. Nevertheless, we performed a theoretical analysis, in which we assembled a minimal set of 11 epitopes suggested restricted by 11 different HLA class I alleles. We then calculated the population coverage, if one was to use this set of epitopes for immunising populations in different areas of the World. We found very high population coverage. The population coverage would be even higher, if we had also considered HLA class I alleles that bind the epitopes as strong as or stronger than the restricting HLA class I allele identified in the present study. Although our discovery of WNV epitopes is based on relatively few patients and could be strengthened by further immunological follow-up experiments, the results indicate that very few epitopes are sufficient for covering the majority of the human population. In the context of an epitope based vaccine against WNV, a larger set of epitopes is, however, preferable to prevent the virus from producing escape variants not containing any of the epitopes. The final composition of an epitope based WNV vaccine in terms of, e.g., subdominant contra dominant epitopes, adjuvant and CD4^+^ T cell epitopes is not dealt with in this study, but clearly these issues also need to be resolved before a vaccine can become a reality.

In conclusion, using advanced bioinformatics methods for CD8^+^ T cell epitope prediction, we have discovered 26 new WNV epitopes that we suggest are restricted by 11 different HLA class I alleles. These epitopes contribute to our knowledge of the immune response against WNV infection and extend the list of known WNV CD8^+^ T cell epitopes.

## Supporting Information

Figure S1 Location of the selected, predicted CD8^+^ T cell epitopes. The 192 selected, predicted epitopes are listed under the reference sequence with RefSeq ID: NC_001563. The HLA class I supertype restriction is listed in parenthesis after the sequence of the epitope. Please note that 17 of the epitopes are predicted to be restricted by more than one HLA class I allele, resulting in a total of 175 unique peptides.
(0.03 MB PDF)

Figure S2 *In vitro* expansion prior to analysis increases sensitivity and does not impact epitope hierarchy. Cryopreserved PBMC from patient #55302 were thawed and rested overnight prior to stimulation for ICS assay (upper panels). A portion of the thawed cells were also subjected to a round of *in vitro* expansion using K64-4-1BBL cells as described the subsection ICS validations of Materials and Methods prior to analysis by ICS assay (lower panels). The numbers reflect the percentage of IFN-γ-positive cells of total live lymphocytes.
(1.28 MB TIF)

Table S1 Measured binding affinity. Of the 175 predicted CD8^+^ T cell epitopes, 161 were synthesised and their in vitro binding affinity to the predicted restricting HLA class I allele was measured. The table lists the 112 peptides that experience a KD below 500 nM.
(0.01 MB PDF)

Table S2 The 26 identified WNV CD8+ T cell epitopes. The columns lists: Sequence: Amino acid sequence of the epitope, Selecting HLA: The HLA class I allele used for selecting the epitope, Protein: Source protein of the epitope, Position: Starting position of the epitope in the source protein, Conservation: Conservation of the epitope in 140 fully sequenced WNV strains obtained from (Koo et al., 2009), Number of responses: The number of responses that were observed against this epitope in this study, Responders: The patients that responded against this epitope. The HLA alleles of each patient are written in subscript after patient ID number. HLA alleles marked in bold are alleles by which the epitope is predicted to be restricted in this patient (see the paragraph “Suggested HLA class I restriction and Table 3 for details), Figure: The figure that illustrates the response.
(0.01 MB PDF)
