# Supplementary material for: Identification of CD8+ T Cell Epitopes in the West Nile Virus Polyprotein by Reverse-Immunology Using NetCTL
Source: PLoS One. 2010 Sep 14;5(9):e12697. doi: 10.1371/journal.pone.0012697 (PMC2939062; doi:10.1371/journal.pone.0012697)
Supplement: Figure S1 — Location of the selected, predicted CD8+ T cell epitopes. The 192 selected, predicted epitopes are listed under the reference sequence with RefSeq ID: NC_001563. The HLA class I supertype restriction is listed in parenthesis after the sequence of the epitope. Please note that 17 of the epitopes are predicted to be restricted by more than one HLA class I allele, resulting in a total of 175 unique peptides. (0.03 MB PDF) [file pone.0012697.s001.pdf]

```
                   10                                                60
NC_001563: MSKKPGGPGKNRAVNMLKRGMPRGLSLIGLKRAMLSLIDGKGPIRFVLALLAFFRFTAIA
                                                  GRGPIRFVL(B39)
                                                         VLALLAFFR(A3)
                                                                    AIAPTRAVL(B7)
                                                       RFVLALLAF(A24)
                                                        FVLALLAFF(A26)
                                  VLSLIGLKR(A3)
                                                    GPIRFVLAL(B7)
                   70                                               120
NC_001563: PTRAVLDRWRGVNKQTAMKHLLSFKKELGTLTSAINRRSTKQKKRGGTAGFTILLGLIAC
                                                               VMIGLIASV(A2)
                     GVNKQTAMK(A3)
                  130                                               180
NC_001563: AGAVTLSNFQGKVMMTVNATDVTDVITIPTAAGKNLCIVRAMDVGYLCEDTITYECPVLA
                                                        FLCDDTITY(B62)
                                                        YLCEDTITY(B62)
                                      IPTAAGKNL(B7)
                  190                                               240
NC_001563: AGNDPEDIDCWCTKSSVYVRYGRCTKTRHSRRSRRSLTVQTHGESTLANKKGAWLDSTKA
                                       HSRRSRRSL(B7)
                                         RRSRRSLTV(B27)
                  250                                               300
NC_001563: TRYLVKTESWILRNPGYALVAAVIGWMLGSNTMQRVVFAILLLLVAPAYSFNCLGMSNRD
                     ILRNPGYAL(B8)
              LVKTESWIL(B8)
            RYLVKTESW(A24)
                                                     LLVAPAYSF(B62)
                                                   LLLLVAPAY(B62)
                  310                                               360
NC_001563: FLEGVSGATWVDLVLEGDSCVTIMSKDKPTIDVKMMNMEAANLADVRSYCYLASVSDLST
                                                                     STKAACPTM(A26)
                  370                                               420
NC_001563: RAACPTMGEAHNEKRADPAFVCKQGVVDRGWGNGCGLFGKGSIDTCAKFACTTKATGWII
                                        GWGNGCGLF(A24)
                  430                                               480
NC_001563: QKENIKYEVAIFVHGPTTVESHGKIGATQAGRFSITPSAPSYTLKLGEYGEVTVDCEPRS
                                                   SYTLKLGEY(A24)
                                            TVSPSAPTY(B62)
                                                                      SGIDTNAYY(A26)
                                                                     RSGIDTNAY(A1)
                  490                                               540
NC_001563: GIDTSAYYVMSVGEKSFLVHREWFMDLNLPWSSAGSTTWRNRETLMEFEEPHATKQSVVA
                               REWFMDLNL(B44)
                         KSFLVHREW(B58)
                             VHREWFMDL(B39)
                  550                                               600
NC_001563: LGSQEGALHQALAGAIPVEFSSNTVKLTSGHLKCRVKMEKLQLKGTTYGVCSKAFKFART
                                                  KLQLKGTTY(A3)
                                                  KLKLKGTTY(A3)
                                                         TYGVCSKAF(A24)
                                                         TYGVCAKAF(A24)
              QEGALHQAL(B44)
                                           KCRVKMEKL(B8)
                  610                                               660
NC_001563: PADTGHGTVVLELQYTGTDGPCKVPISSVASLNDLTPVGRLVTVNPFVSVATANSKVLIE
                                                 GRLVTVNPF(B27)
                                  VPISSVASL(B7)
                 GTVVLELQY(A1)
               GHGTVVLEL(B39)
                  670                                               720
NC_001563: LEPPFGDSYIVVGRGEQQINHHWHKSGSSIGKAFTTTLRGAQRLAALGDTAWDFGSVGGV
                                                      LAALGDTAW(B58)
                  730                                               780
NC_001563: FTSVGKAIHQVFGGAFRSLFGGMSWITQGLLGALLLWMGINARDRSIAMTFLAVGGVLLF
                           RSLFGGMSW(B58)
                                GMSWITQGL(A2)
                            SLFGGMSWI(A2)
                                                             FLAVGGVLL(A2)
                  790                                               840
NC_001563: LSVNVHADTGCAIDIGRQELRCGSGVFIHNDVEAWMDRYKFYPETPQGLAKIIQKAHAEG
                                                   YHPETPQGL(B39)
                                                      ETPQGLAKI(A26)
                  850                                               900
NC_001563: VCGLRSVSRLEHQMWEAIKDELNTLLKENGVDLSVVVEKQNGMYKAAPKRLAATTEKLEM
                                                    GLYKSAPRR(A3)
                                                           RRLAATTEK(B27)
                                              VVEKQSGLY(A1)
                                     KENGVDLSV(B44)
                 VSRLEHQMW(B58)
```

```
                  910                                               960
NC_001563: GWKAWGKSIIFAPELANNTFVIDGPETEECPTANRAWNSMEVEDFGFGLTSTRMFLRIRE
             KAWGKSIIF(B58)
                  970                                              1020
NC_001563: TNTTECDSKIIGTAVKNNMAVHSDLSYWIESGLNDTWKLERAVLGEVKSCTWPETHTLWG
                 1030                                              1080
NC_001563: DGVLESDLIIPITLAGPRSNHNRRPGYKTQNQGPWDEGRVEIDFDYCPGTTVTISDSCEH
                                                        YCPGTTVTL(B39)
                 1090                                              1140
NC_001563: RGPAARTTTESGKLITDWCCRSCTLPPLRFQTENGCWYGMEIRPTRHDEKTLVQSRVNAY
                                        YQTDSGCWY(A1)
                                               WYGMEIRPL(A24)
                 1150                                              1200
NC_001563: NADMIDPFQLGLMVVFLATQEVLRKRWTAKISIPAIMLALLVLVFGGITYTDVLRYVILV
                                                          ITYTDVLRY(A1)
                                VLRKRWTAK(A3)
                  FQLGLLVVF(B27)
                                                                  YVILVGAAF(A26)
                 1210                                              1260
NC_001563: GAAFAEANSGGDVVHLALMATFKIQPVFLVASFLKARWTNQESILLMLAAAFFQMAYYDA
                                   QPAFMVASF(B7)
                                                      ILLMLAAAF(B62)
                                                         MLAAAFFQM(A2)
                                   QPVFMVASF(B7)
                 1270                                              1320
NC_001563: KNVLSWEVPDVLNSLSVAWMILRAISFTNTSNVVVPLLALLTPGLKCLNLDVYRILLLMV
             ILLWEIPDV(A2)
                                                                     MVGVGSLVK(A3)
                             WMILRAISF(A24)
                 EIPDVLNSL(A26)
                 1330                                              1380
NC_001563: GVGSLIKEKRSSAAKKKGACLICLALASTGVFNPMILAAGLMACDPNRKRGWPATEVMTA
                                                                 TEVMTAVGL(B44)
                                                                  EVMTAVGLM(A26)
                       AAKKKGASL(B7)
                 1390                                              1440
NC_001563: VGLMFAIVGGLAELDIDSMAIPMTIAGLMFAAFVISGKSTDMWIERTADITWESDAEITG
                AIVGGLAEL(A2)
                                             ISGKSTDMW(B58)
                 1450                                              1500
NC_001563: SSERVDVRLDDDGNFQLMNDPGAPWKIWMLRMACLAISAYTPWAILPSVIGFWITLQYTK
                                             LAVSAYTPW(B58)
                                GAPWKIWML(B8)
                                             LAISAYTPW(B58)
                           LMNDPGAPW(B62)
                 1510                                              1560
NC_001563: RGGVLWDTPSPKEYKKGDTTTGVYRIMTRGLLGSYQAGAGVMVEGVFHTLWHTTKGAALM
              VLWDTPSPK(A3)
                                  YRIMTRGLL(B27)
                                     MTRGLLGSY(A1)
                                                             WHTTKGAAL(B39)
                                                              HTTKGAALM(A26)
                                     MTRGILGSY(A1)
                          KGDTTTGVY(A1)
                                 VYRIMTRGL(A24)
                 1570                                              1620
NC_001563: SGEGRLDPYWGSVKEDRLCYGGPWKLQHKWNGHDEVQMIVVEPGKNVKNVQTKPGVFKTP
                           RLCYGGPWK(A3)
                 1630                                              1680
NC_001563: EGEIGAVTLDYPTGTSGSPIVDKNGDVIGLYGNGVIMPNGSYISAIVQGERMEEPAPAGF
                                                              MEEPAPAGF(B44)
                                            GVIMPNGSY(B62)
                 1690                                              1740
NC_001563: EPEMLRKKQITVLDLHPGAGKTRKILPQIIKEAINKRLRTAVLAPTRVVAAEMSEALRGL
              MLRKKQITV(B8)
                 1750                                              1800
NC_001563: PIRYQTSAVHREHSGNEIVDVMCHATLTHRLMSPHRVPNYNLFIMDEAHFTDPASIAARG
                                                                      GYISTRVEL(A24)
                     REHSGNEIV(B44)
                                          MSPHRVPNY(A1)
                                 CHATLTHRL(B39)
                 1810                                              1860
NC_001563: YIATKVELGEAAAIFMTATPPGTSDPFPESNAPISDMQTEIPDRAWNTGYEWITEYVGKT
                                                                 EYVGKTVWF(A24)
                                                      RAWNSGYEW(B58)
                 1870                                              1920
NC_001563: VWFVPSVKMGNEIALCLQRAGKKVIQLNRKSYETEYPKCKNDDWDFVITTDISEMGANFK
                                        KSYETEYPK(A3)
                                  VIQLNRKSY(B62)
                 1930                                              1980
```

```
NC_001563: ASRVIDSRKSVKPTIIEEGDGRVILGEPSAITAASAAQRRGRIGRNPSQVGDEYCYGGHT
                           TEGEGRVIL(B39)
                                                                     HTNEDDSNF(A26)
             RVIDSRKSV(B7)
                 1990                                              2040
NC_001563: NEDDSNFAHWTEARIMLDNINMPNGLVAQLYQPEREKVYTMDGEYRLRGEERKNFLEFLR
                                                            EERKNFLEL(B44)
                                               KVYTMDGEY(A3)
                                                 YTMDGEYRL(A2)
                                                       YRLRGEERK(B27)
                                         YQPEREKVY(B62)
                 2050                                              2100
NC_001563: TADLPVWLAYKVAAAGISYHDRKWCFDGPRTNTILEDNNEVEVITKLGERKILRPRWADA
                                                                RPRWADARV(B7)
                     KVAAAGVSY(B62)
                                                 NEVEVITKL(B44)
                            SYHDRRWCF(A24)
                                RRWCFDGPR(B27)
                 2110                                              2160
NC_001563: RVYSDHQALKSFKDFASGKRSQIGLVEVLGRMPEHFMVKTWEALDTMYVVATAEKGGRAH
                                                   WEALDTMYV(B44)
                                                                  GGRAHRMAL(B7)
           RVYSDHQAL(B7)
                 2170                                              2220
NC_001563: RMALEELPDALQTIVLIALLSVMSLGVFFLLMQRKGIGKIGLGGVILGAATFFCWMAEVP
                                VMTMGVFFL(A2)
                                                         LGAATFFCW(B58)
                                    GVFFLLMQR(A3)
                 2230                                              2280
NC_001563: GTKIAGMLLLSLLLMIVLIPEPEKQRSQTDNQLAVFLICVLTLVGAVAANEMGWLDKTKN
                                      QTDNQLAVF(A1)
                 2290                                              2340
NC_001563: DIGSLLGHRPEARETTLGVESFLLDLRPATAWSLYAVTTAVLTPLLKHLITSDYINTSLT
                                           SLYAVTTAV(A2)
                                            LYAVTTAVL(A24)
                       RESFGVESF(B44)
                                                             TSDYINTSL(B39)
                 2350                                              2400
NC_001563: SINVQASALFTLARGFPFVDVGVSALLLAVGCWGQVTLTVTVTAAALLFCHYAYMVPGWQ
                     SLARGFPFV(A2)
                     TLARGFPFV(A2)
                            FVDVGVSAL(B39)
                 2410                                              2460
NC_001563: AEAMRSAQRRTAAGIMKNVVVDGIVATDVPELERTTPVMQKKVGQIILILVSMAAVVVNP
                   RRTAAGIMK(B27)
               RAAQRRTAA(B7)
                 2470                                              2520
NC_001563: SVRTVREAGILTTAAAVTLWENGASSVWNATTAIGLCHIMRGGWLSCLSIMWTLIKNMEK
                      ITAAAVTLW(B58)
                 2530                                              2580
NC_001563: PGLKRGGAKGRTLGEVWKERLNHMTKEEFTRYRKEAITEVDRSAAKHARREGNITGGHPV
                                  LTKEEFTRY(A1)
                                       FTRYRKEAI(B8)
                                  MTKEEFTRY(A1)
                 2590                                              2640
NC_001563: SRGTAKLRWLVERRFLEPVGKVVDLGCGRGGWCYYMATQKRVQEVKGYTKGGPGHEEPQL
                 LRWLVERRF(B27)
                       RRFLEPVGK(B27)
                                                  KRVQEVRGY(B27)
                                                  KRVQEVKGY(B27)
                 2650                                              2700
NC_001563: VQSYGWNIVTMKSGVDVFYRPSEASDTLLCDIGESSSSAEVEEHRTVRVLEMVEDWLHRG
                                                                  WLHRGPKEF(B8)
                                                 AEVEEHRTV(B44)
                                                          RVLEMVEDW(B58)
                 2710                                              2760
NC_001563: PKEFCIKVLCPYMPKVIEKMETLQRRYGGGLIRNPLSRNSTHEMYWVSHASGNIVHSVNM
                                   RRYGGGLVR(B27)
                 KVLCPYMPK(A3)
                         KVIEKMEVL(B8)
                  VLCPYMPKV(A2)
              FCIKVLCPY(A26)
                 2770                                              2820
NC_001563: TSQVLLGRMEKKTWKGPQFEEDVNLGSGTRAVGKPLLNSDTSKIKNRIERLKKEYSSTWH
                                       TRAVGKPLL(B39)
                                                     KIRNRIERL(B8)
                           AHYEEDVNL(B39)
                 GRMEKKTWK(B27)
                                                     KIKNRIERL(B8)
                 2830                                              2880
```

```
NC_001563: QDANHPYRTWNYHGSYEVKPTGSASSLVNGVVRLLSKPWDTITNVTTMAMTDTTPFGQQR
                                                  DTITNVTTM(A26)
                  RTWNYHGSY(A1)
                                    SLVNGVVRL(A2)
                             KPTGSASSL(B7)
                 2890                                              2940
NC_001563: VFKEKVDTKAPEPPEGVKYVLNETTNWLWAFLARDKKPRMCSREEFIGKVNSNAALGAMF
                                 ETTNWLWTF(A26)
                                                                    AMFEEQNQW(B62)
                              VLNETTNWL(A2)
                                 ETTNWLWAF(A26)
                 2950                                              3000
NC_001563: EEQNQWKNAREAVEDPKFWEMVDEEREAHLRGECNTCIYNMMGKREKKPGEFGKAKGSRA
                                                                    SRAIWFMWL(B27)
                                                                   GSRAIWFMW(B58)
                 3010                                              3060
NC_001563: IWFMWLGARFLEFEALGFLNEDHWLGRKNSGGGVEGLGLQKLGYILKEVGTKPGGKVYAD
               WLGARFLEF(A24)
                                            VEGLGLQKL(B44)
            WFMWLGARF(A24)
                     LEFEALGFL(B44)
                 3070                                              3120
NC_001563: DTAGWDTRITKADLENEAKVLELLDGEHRRLARSIIELTYRHKVVKVMRPAADGKTVMDV
                                                           RPAVGGKTV(B7)
                                                  YRHKVVKVM(B27)
                                                           RPAADGKTV(B7)
                                        RLARAIIEL(A2)
                          NEAKVLELL(B44)
                                                LTYRHKVVK(A3)
                 3130                                              3180
NC_001563: ISREDQRGSGQVVTYALNTFTNLAVQLVRMMEGEGVIGPDDVEKLGKGKGPKVRTWLFEN
                                                            GPKVRTWLF(B8)
                                                            GVKVRVWLF(B8)
                 3190                                              3240
NC_001563: GEERLSRMAVSGDDCVVKPLDDRFATSLHFLNAMSKVRKDIQEWKPSTGWYDWQQVPFCS
                                                            WYDWQQVPF(A24)
                 3250                                              3300
NC_001563: NHFTELIMKDGRTLVVPCRGQDELIGRARISPGAGWNVRDTACLAKSYAQMWLLLYFHRR
                                                                  YFHRRDLRL(B8)
                                                  DTACLAKSY(A1)
                                                          YAQMWQLLY(A1)
                                                        KSYAQMWLL(B58)
                                                          YAQMWLLLY(A1)
                 3310                                              3360
NC_001563: DLRLMANAICSAVPANWVPTGRTTWSIHAKGEWMTTEDMLAVWNRVWIEENEWMEDKTPV
                                                       RVWIEENEW(B58)
                 3370                                              3420
NC_001563: ERWSDVPYSGKREDIWCGSLIGTRTRATWAENIHVAINQVRSVIGEEKYVDYMSSLRRYE
                      REDIWCGSL(B44)
                 3430
NC_001563: DTIVVEDTVL
```
