# Supplementary figures and images for: Identification of CD8+ T Cell Epitopes in the West Nile Virus Polyprotein by Reverse-Immunology Using NetCTL
Source: PLoS One. 2010 Sep 14;5(9):e12697. doi: 10.1371/journal.pone.0012697 (PMC2939062; doi:10.1371/journal.pone.0012697)

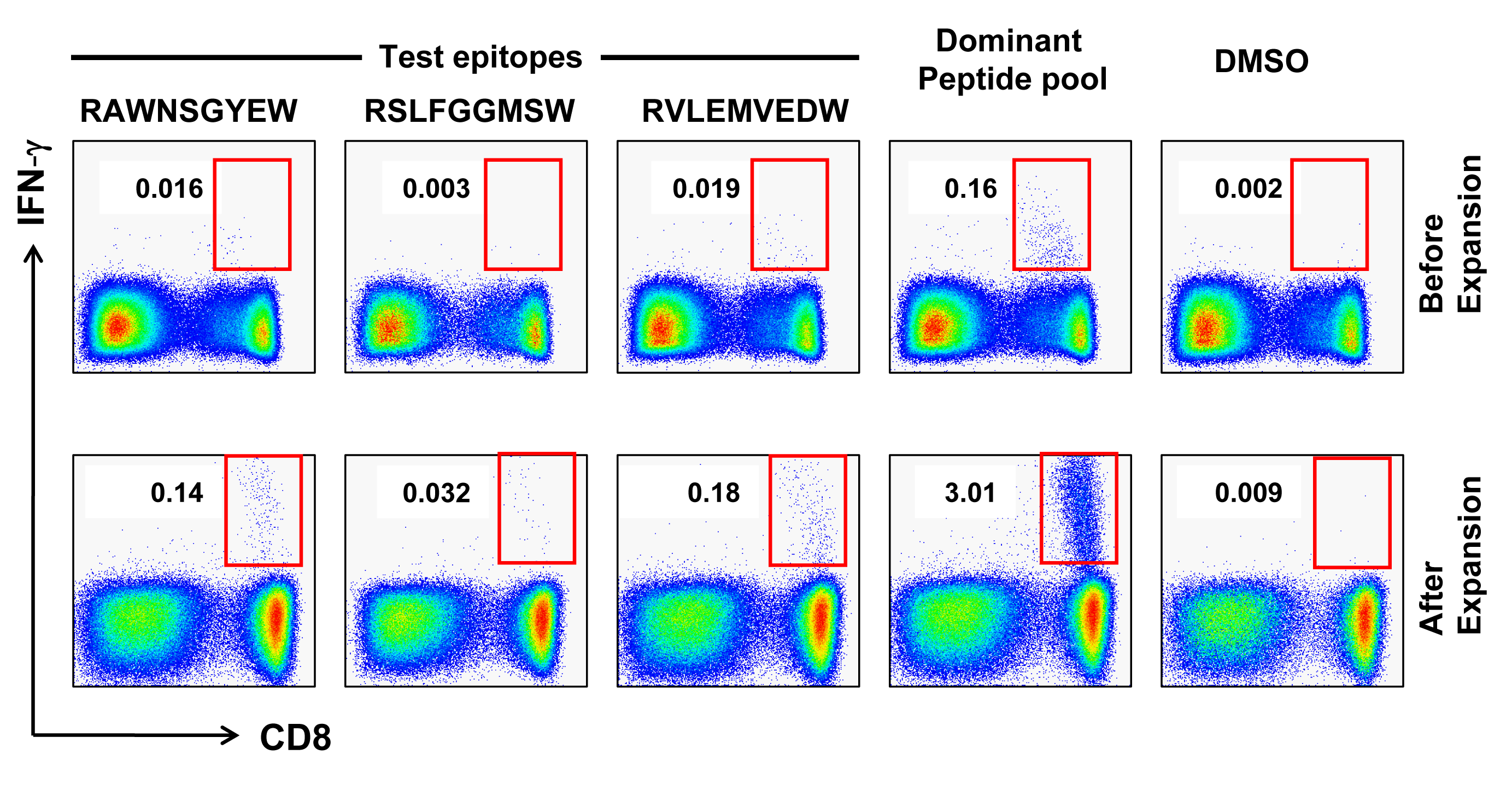

Supplement: Figure S2 — In vitro expansion prior to analysis increases sensitivity and does not impact epitope hierarchy. Cryopreserved PBMC from patient #55302 were thawed and rested overnight prior to stimulation for ICS assay (upper panels). A portion of the thawed cells were also subjected to a round of in vitro expansion using K64-4-1BBL cells as described the subsection ICS validations of Materials and Methods prior to analysis by ICS assay (lower panels). The numbers reflect the percentage of IFN-γ-positive cells of total live lymphocytes. (1.28 MB TIF) [file pone.0012697.s002.tif]
