# Supplementary material for: Identification of CD8+ T Cell Epitopes in the West Nile Virus Polyprotein by Reverse-Immunology Using NetCTL
Source: PLoS One. 2010 Sep 14;5(9):e12697. doi: 10.1371/journal.pone.0012697 (PMC2939062; doi:10.1371/journal.pone.0012697)
Supplement: Table S1 — Measured binding affinity. Of the 175 predicted CD8+ T cell epitopes, 161 were synthesised and their in vitro binding affinity to the predicted restricting HLA class I allele was measured. The table lists the 112 peptides that experience a KD below 500 nM. (0.01 MB PDF) [file pone.0012697.s003.pdf]

Sequence	HLA	Measured K_D (nM)
**ITYTDVLRY**	A*0101	3
**VVEKQSGLY**	A*0101	6
**RSGIDTNAY**	A*0101	66
**QTDNQLAVF**	A*0101	168
**RTWNYHGSY**	A*0101	237
**MTKEEFTRY**	A*0101	246
**KGDTTTGVY**	A*0101	472
**SLFGGMSWI**	A*0201	1
**GMSWITQGL**	A*0201	1
**ILLWEIPDV**	A*0201	1
**VLNETTNWL**	A*0201	2
**FLAVGGVLL**	A*0201	3
**RLARAIIEL**	A*0201	3
**TLARGFPFV**	A*0201	3
**SLVNGVVRL**	A*0201	5
**VLCPYMPKV**	A*0201	21
**GLYKSAPRR**	A*0301	37
**VLRKRWTAK**	A*0301	43
**LTYRHKVVK**	A*0301	94
**VLWDTPSPK**	A*0301	117
**MVGVGSLVK**	A*0301	380
**VLSLIGLKR**	A*0301	481
**RYLVKTESW**	A*2403	4
**WYDWQQVPF**	A*2403	7
**LYAVTTAVL**	A*2403	8
**SYHDRRWCF**	A*2403	9
**WFMWLGARF**	A*2403	10
**TYGVCAKAF**	A*2403	13
**TYGVCSKAF**	A*2403	25
**EYVGKTVWF**	A*2403	26
**GYISTRVEL**	A*2403	45
**WMILRAISF**	A*2403	120
**SYTLKLGEY**	A*2403	143
**RFVLALLAF**	A*2403	176
**ETTNWLWAF**	A*2601	2
**DTITNVTTM**	A*2601	4
**EVMTAVGLM**	A*2601	5
**ETTNWLWTF**	A*2601	5
**MTRGLLGSY**	A*2601	8
**DTACLAKSY**	A*2601	8

**HTTKGAALM**	A*2601	23
**FCIKVLCPY**	A*2601	314
**AIAPTRAVL**	B*0702	2
**RPRWADARV**	B*0702	9
**RAAQRRTAA**	B*0702	11
**GPIRFVLAL**	B*0702	13
**AAKKKGASL**	B*0702	15
**VPISSVASL**	B*0702	25
**KPTGSASSL**	B*0702	27
**RPAVGGKTV**	B*0702	36
**HSRRSRRSL**	B*0702	47
**GGRAHRMAL**	B*0702	63
**QPAFMVASF**	B*0702	71
**RVIDSRKSV**	B*0702	97
**RPAADGKTV**	B*0702	97
**IPTAAGKNL**	B*0702	150
**RVYSDHQAL**	B*0702	205
**MLRKKQITV**	B*0801	35
**FTRYRKEAI**	B*0801	39
**GPKVRTWLF**	B*0801	126
**KIRNRIERL**	B*0801	254
**ILRNPGYAL**	B*0801	483
**AMFEEQNQW**	B*1501	17
**GVIMPNGSY**	B*1501	29
**LMNDPGAPW**	B*1501	40
**TVSPSAPTY**	B*1501	68
**YQPEREKVY**	B*1501	80
**LLLLVAPAY**	B*1501	87
**VIQLNRKSY**	B*1501	105
**LLVAPAYSF**	B*1501	137
**KVAAAGVSY**	B*1501	165
**YLCEDTITY**	B*1501	197
**RRWCFDGPR**	B*2705	11
**RRTAAGIMK**	B*2705	24
**YRHKVVKVM**	B*2705	33
**YRIMTRGLL**	B*2705	35
**RRLAATTEK**	B*2705	37
**RRFLEPVGK**	B*2705	50
**RRYGGGLVR**	B*2705	62
**GRLVTVNPF**	B*2705	75
**LRWLVERRF**	B*2705	98
**GRMEKKTWK**	B*2705	103

**RRSRRSLTV**	B*2705	385
**WHTTKGAAL**	B*3901	17
**FVDVGVSAL**	B*3901	52
**CHATLTHRL**	B*3901	64
**TRAVGKPLL**	B*3901	141
**YTMDGEYRL**	B*3901	154
**GRGPIRFVL**	B*3901	162
**RESFGVESF**	B*4001	1
**REHSGNEIV**	B*4001	1
**KENGVDLSV**	B*4001	3
**REDIWCGSL**	B*4001	7
**REWFMDLNL**	B*4001	9
**TEVMTAVGL**	B*4001	10
**QEGALHQAL**	B*4001	14
**WEALDTMYV**	B*4001	27
**NEAKVLELL**	B*4001	28
**NEVEVITKL**	B*4001	80
**AEVEEHRTV**	B*4001	130
**VEGLGLQKL**	B*4001	230
**RVLEMVEDW**	B*5801	4
**RSLFGGMSW**	B*5801	5
**KSYAQMWLL**	B*5801	8
**LAVSAYTPW**	B*5801	11
**KSFLVHREW**	B*5801	11
**RAWNSGYEW**	B*5801	16
**KAWGKSIIF**	B*5801	42
**VSRLEHQMW**	B*5801	56
**LAALGDTAW**	B*5801	66
**GSRAIWFMW**	B*5801	87
**ISGKSTDMW**	B*5801	91
