# Supplementary material for: Identification of CD8+ T Cell Epitopes in the West Nile Virus Polyprotein by Reverse-Immunology Using NetCTL
Source: PLoS One. 2010 Sep 14;5(9):e12697. doi: 10.1371/journal.pone.0012697 (PMC2939062; doi:10.1371/journal.pone.0012697)
Supplement: Table S2 — The 26 identified WNV CD8+ T cell epitopes. The columns lists: Sequence: Amino acid sequence of the epitope, Selecting HLA: The HLA class I allele used for selecting the epitope, Protein: Source protein of the epitope, Position: Starting position of the epitope in the source protein, Conservation: Conservation of the epitope in 140 fully sequenced WNV strains obtained from (Koo et al., 2009), Number of responses: The number of responses that were observed against this epitope in this study, Responders: The patients that responded against this epitope. The HLA alleles of each patient are written in subscript after patient ID number. HLA alleles marked in bold are alleles by which the epitope is predicted to be restricted in this patient (see the paragraph “Suggested HLA class I restriction and Table 3 for details), Figure: The figure that illustrates the response. (0.01 MB PDF) [file pone.0012697.s004.pdf]

Sequence	Selecting HLA	Protein	Pos.	Cons. (%)	No. of responses	Responders	Figure
YTMDGEYRL	B*3901	NS3	518	97	3	55410 **A*0201-A*0201**-B*4001-B*4402-Cw*0304-Cw*0501<br>55413 A*0101-**A*0201**-B*0801-B*4402-Cw*0701-Cw*0501<br>55309 **A*0201**-A*0301-B*3503-B*4403-Cw*0401-Cw*0401	1
RYLVKTESW	A*2403	prM	119	99	2	55410 A*0201-A*0201-B*4001-B*4402-Cw*0304-Cw*0501<br>55415 A*0201-A*0201-**B*2702-**B*5601-Cw*0102-Cw*0202	1
LTYRHKVVK	A*0301	NS5	573	100	2	55309 A*0201-**A*0301**-B*3503-B*4403-Cw*0401-Cw*0401<br>55308 A*0101-**A*0301**-B*0801-B*4701-Cw*0602-Cw*0701	1
GPIRFVLAL	B*0702	C	42	96	1	55405 A*0101-A*0301-**B*0702**-B*0801-Cw*0701-Cw*0702	1
TEVMTAVGL	B*4001	NS2B	5	99	1	55410 A*0201-A*0201-**B*4001**-B*4402-Cw*0304-Cw*0501	1
ILRNPGYAL	B*0801	prM	128	100	1	55410 A*0201-A*0201-B*4001-B*4402-**Cw*0304**-Cw*0501	1
YRHKVVKVM	B*2705	NS5	575	100	1	55307 A*0101-A*0301-B*3701-B*4429-Cw*0501-**Cw*0602**	1
SYHDRRWCF	A*2403	NS3	557	89	1	55405 A*0101-A*0301-B*0702-**B*0801**-Cw*0701-Cw*0702	1
AEVEEHRTV	B*4001	NS5	154	8	1	55309 A*0201-A*0301-B*3503-**B*4403**-Cw*0401-Cw*0401	1
GLYKSAPRR	A*0301	NS1	95	1	1	55415 A*0201-A*0201-B*2702-B*5601-Cw*0102-Cw*0202	1
KGDTTTGVY	A*0101	NS3	15	96	2	55410 A*0201-A*0201-B*4001-B*4402-Cw*0304-Cw*0501<br>55415 A*0201-A*0201-B*2702-B*5601-Cw*0102-Cw*0202	1/2
RPAADGKTV	B*0702	NS5	584	8	1	55415 A*0201-A*0201-B*2702-**B*5601**-Cw*0102-Cw*0202	1
HTTKGAALM	A*2601	NS3	51	100	1	55309 A*0201-A*0301-B*3503-B*4403-Cw*0401-Cw*0401	1
RRSRRSLTV	B*2705	prM	88	99	1	55405 A*0101-A*0301-B*0702-**B*0801**-Cw*0701-Cw*0702	1
FVDVGVSAL	B*3901	NS4B	112	99	1	55405 A*0101-A*0301-**B*0702**-B*0801-Cw*0701-Cw*0702	1
RAWNSGYEW	B*5801	NS3	343	94	1	55302 A*01-A*01-**B*57**-B*40	1
RSLFGGMSW	B*5801	E	447	99	1	55302 A*01-A*01-**B*57**-B*40	1
RVLEMVEDW	B*5801	NS5	163	99	1	55302 A*01-A*01-**B*57**-B*40	1

VLNETTNWL	A*0201	NS5	375	100	2	44401 A*0101-**A*0201**-B*0702-B*1517-Cw*0701-Cw*0702<br>55410 **A*0201-A*0201**-B*4001-B*4402-Cw*0304-Cw*0501	2
SLVNGVVRL	A*0201	NS5	321	100	4	44405 A*0101-**A*0201**-B*0702-B*1501-Cw*0303-Cw*0702<br>55410 **A*0201-A*0201**-B*4001-B*4402-Cw*0304-Cw*0501<br>55415 **A*0201-A*0201**-B*2702-B*5601-Cw*0102-Cw*0202<br>55413 A*0101-**A*0201**-B*0801-B*4402-Cw*0701-Cw*0501	2
SLFGGMSWI	A*0201	E	448	99	1	44401 A*0101-**A*0201**-B*0702-B*1517-Cw*0701-Cw*0702	2
TLARGFPFV	A*0201	NS4B	105	96	1	55413 A*0101-**A*0201**-B*0801-B*4402-Cw*0701-Cw*0501	2
ILLWEIPDV	A*0201	NS2A	124	87	2	44401 A*0101-**A*0201**-B*0702-B*1517-Cw*0701-Cw*0702<br>55405 A*0101-A*0301-B*0702-B*0801-Cw*0701-Cw*0702	1/2
MTKEEFTRY	A*0101	NS5	19	94	1	44405 **A*0101**-A*0201-B*0702-B*1501-Cw*0303-Cw*0702	2
VVEKQSGLY	A*0101	NS1	89	1	1	44401 **A*0101**-A*0201-B*0702-B*1517-Cw*0701-Cw*0702	2
ITYTDVLRY	A*0101	NS2A	49	98	1	44405 **A*0101**-A*0201-B*0702-B*1501-Cw*0303-Cw*0702	2
